# Single base substitution and insertion/deletion mutational signatures in adult core binding factor acute myeloid leukemia

**DOI:** 10.1038/s41375-022-01552-x

**Published:** 2022-04-01

**Authors:** Rebeqa Gunnarsson, Minjun Yang, Andrea Biloglav, Vladimir Lazarevic, Kajsa Paulsson, Bertil Johansson

**Affiliations:** 1 Division of Clinical Genetics, Department of Laboratory Medicine, Lund University, Lund, Sweden; 2 Department of Hematology, Oncology and Radiation Physics, Skåne University Hospital, Lund, Sweden; 3 Stem Cell Centre, Lund University, Lund, Sweden; 4 Department of Clinical Genetics and Pathology, Office for Medical Services, Region Skåne, Lund, Sweden

**Keywords:** Cancer genomics, Translational research, Cancer genomics

## To the Editor

Single base substitutions (SBSs) and insertions/deletions (indels; IDs) arise through several mechanisms such as errors during DNA replication/repair and exposures to mutagens, with the different mutational processes occasionally generating specific mutational signatures. SBS signatures (SBSsigns) result from recurring trinucleotide patterns of the transition/transversion types of somatic single nucleotide variants (SNVs) and their flanking nucleotides, whereas ID signatures (IDsigns) are defined according to size, nucleotides affected, and the presence of repetitive/microhomology regions (https://cancer.sanger.ac.uk/signatures/). Some signatures are associated with underlying etiologic factors, e.g. SBS7 and ID13 in UV-associated melanoma and SBS4 and ID3 in smoking-induced lung cancer [1, 2], whereas others are linked to inherent defects of DNA recombination, replication, and repair (SBS6 and ID1) or caused by spontaneous or enzymatic deamination of 5-methylcytosine to thymine (SBS1) (https://cancer.sanger.ac.uk/signatures/).

Based on whole genome sequencing (WGS) of pediatric acute myeloid leukemia (AML), we recently reported that SBS18 – a signature characterized by frequent C > A transversions – is enriched in t(8;21)(q22;q22)/*RUNX1*::*RUNX1T1*-positive cases [3] (gene fusion designation according to recent guidelines [4]). Brandsma et al. [5] subsequently also showed that SBS18 is common in childhood AML, including cases with *RUNX1*::*RUNX1T1*. Considering that SBS18 has been associated with DNA damage caused by reactive oxygen species (ROS) (https://cancer.sanger.ac.uk/signatures/sbs/sbs18/) and that the RUNX1::RUNX1T1 chimeric protein is known to downregulate the expression of the *OGG1* gene encoding a DNA glycosylase that excises oxidized guanines [6], we hypothesized that ROS could be involved in the genesis of childhood AML with *RUNX1*::*RUNX1T1* [3].

Whether SBS18 is overrepresented also in adult *RUNX1*::*RUNX1T1*-positive AML is unknown. In fact, our knowledge of SBSsigns is rudimentary—and non-existing as regards IDsigns—in adult core binding factor (CBF) AML, which consists of cases positive for either *RUNX1*::*RUNX1T1* or *CBFB*::*MYH11* [inv(16)(p13q22)/t(16;16)(p13;q22)] [7]. The only publication to date addressing SBSsigns in adult CBF AML reported a high frequency of SBS1 [8], a clock-like signature that accumulates with age (https://cancer.sanger.ac.uk/signatures/). To ascertain if SBS18 is a common mutational signature in adult CBF AML, we performed WGS of ten cases with *RUNX1*::*RUNX1T1* and ten with *CBFB*::*MYH11*, focusing not only on SBSsigns but also on IDsigns. All patients had *de novo* AML, thus excluding those previously exposed to chemo- and/or radiotherapy that could have affected the mutational signatures. The cases were selected based on the availability of good quality DNA from both diagnosis and remission. The median age of the patients was 51.5 years (range 19-74 years) and the female/male ratio was 1:1.5. All genetic analyses were performed at the Department of Clinical Genetics and Pathology, Office for Medical Services, Region Skåne, Lund, Sweden. The basic clinical and genetic features of the CBF AMLs are summarized in Supplementary Table 1 and data on WGS of paired diagnostic/remission samples and bioinformatic analyses are provided in Supplementary Information.

The average sequencing depths of the WGS varied from 29× to 57× per sample (median 40×) and the Q30 value was 96.23%, with 2 × 150 bp read length. The WGS analyses confirmed the *RUNX1*::*RUNX1T1* and *CBFB*::*MYH11* gene fusions in all cases and also revealed that the genomic breaks clustered within introns 6 of *RUNX1* and 1 of *RUNX1T1* and within introns 5 of *CBFB* and 33 of *MYH11*, respectively (Supplementary Table 1). No other chimeric genes were detected. All chromosomal gains and losses previously found by conventional G-banding were identified by WGS except for two subclonal trisomies in one case (Supplementary Tables 1 and 2). WGS also identified 32 copy number abnormalities (≤10 Mb) and five uniparental isodisomies, all of which undetectable by chromosome banding analyses (Supplementary Table 2). None of the cases displayed any signs of chromothripsis.

A median of 1437 (range 22–1834) and 1049 (561–1369) SNVs was identified in the *RUNX1*::*RUNX1T1*- and *CBFB*::*MYH11*-positive cases, respectively, corresponding to 0.01-0.61 SNVs/indels per Mb. Comparing the transition and transversion types between the two gene fusion groups revealed highly similar frequency distributions, except for a slight excess of C > T transitions in the cases with *CBFB*::*MYH11* (49% versus 47%; *P* = 0.012; Mann–Whitney U test; Supplementary Fig. 1). Overall, the most common substitution types were, in decreasing order, C > T, T > C, C > A, T > A, C > G, and T > G (Supplementary Fig. 1). The frequencies of all these increased significantly with age (Supplementary Fig. 2), suggesting clock-like acquisitions [9]. The rainfall plot analysis revealed no evidence for any hypermutated (kataegic) regions in the CBF AMLs (data not shown). A median of 79 (range 9–115) and 55 (27–82) indels was detected in the *RUNX1*::*RUNX1T1*- and *CBFB*::*MYH11*-positive cases, respectively (0.003–0.038 indels per Mb). In total, 24,597 SNVs and indels were detected, of which 307 (1.2%) occurred in coding regions. Most of the recurrently mutated genes identified have previously been reported to harbor pathogenic SNVs and indels in CBF AML (Supplementary Table 3), e.g., *ASXL2*, *KIT*, *KRAS*, and *ZBTB7A* [8, 10].

**Fig. 1:**
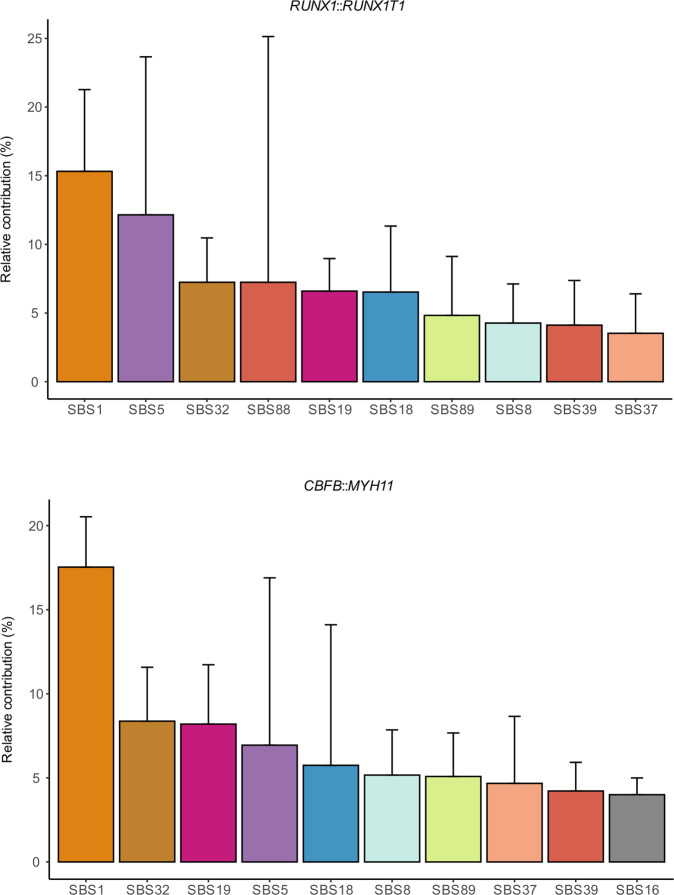
Frequencies of the ten most common single base substitution (SBS) mutational signatures in adult core binding factor acute myeloid leukemia. The signatures are based on their relative contributions per case within each gene fusion group and are shown separately for the *RUNX1*::*RUNX1T1* (upper panel) and *CBFB*::*MYH11* (lower panel) gene fusion groups.

**Fig. 2:**
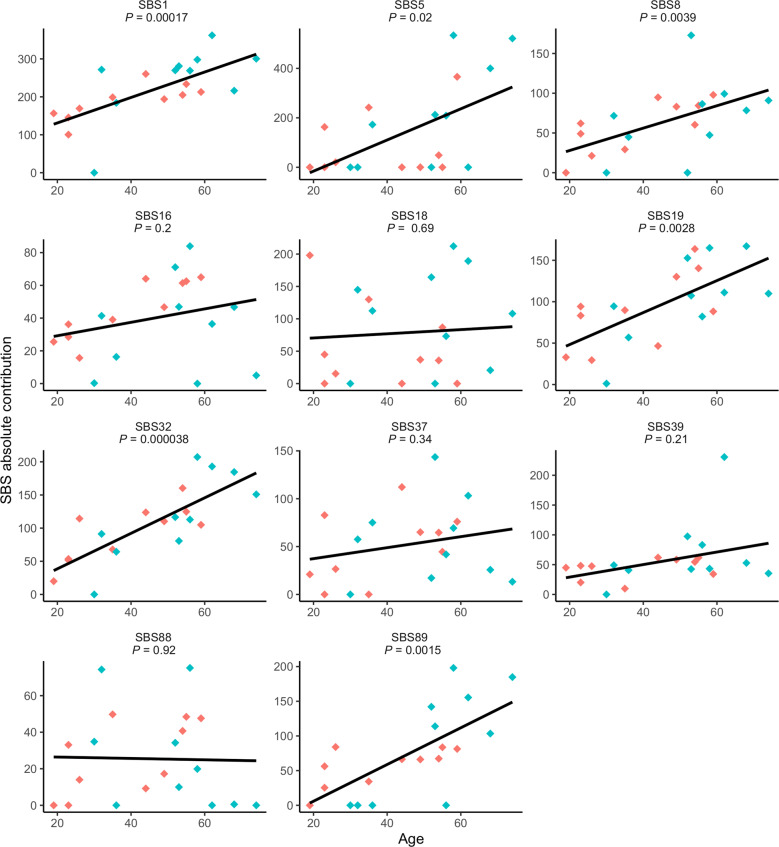
Absolute contributions of the most common single base substitution (SBS) signatures in adult core binding factor acute myeloid leukemia in relation to age. The *RUNX1*::*RUNX1T1*- and *CBFB*::*MYH11*-positive cases are shown in blue and red, respectively. The *P* values are based on linear regression analyses.

To investigate the presence/frequencies of the different SBSsigns and IDsigns in the CBF AMLs, COSMIC v.3.2 (https://cancer.sanger.ac.uk/signatures/) was used. Among the top ten SBSsigns in the cases with *RUNX1*::*RUNX1T1* or *CBFB*::*MYH11*, nine were present in both groups: SBS1 (the most common one), SBS5, SBS8, SBS18, SBS19, SBS32, SBS37, SBS39, and SBS89, albeit with varying frequencies between the two fusion groups (Fig. 1). SBS5 has been associated with smoking in several cancer types, *e.g*., bladder cancer with *ERCC2* mutations (https://cancer.sanger.ac.uk/signatures/sbs/sbs5). The smoking habits of the CBF AML patients were unknown, but none of the cases harbored variants in *ERCC2* (Supplementary Table 3). SBS32 has been linked to prior therapy with azathioprine (https://cancer.sanger.ac.uk/signatures/sbs/sbs32/); however, no patient had received such treatment, indicating that mutational mechanisms other than exposure to azathioprine contribute to SBS32. SBS18 was also among the ten most common SBS signatures in both fusion groups: 1%-13% of the SBSs in eight of the ten cases with *RUNX1*::*RUNX1T1* and in 2%-28% in 7/10 *CBFB*::*MYH11*-positive cases (Fig. 1). However, the relative contribution of SBS18 was significantly higher (18–28% in all cases) in pediatric *RUNX1*::*RUNX1T1*-positive cases in our previous study [3] (Supplementary Fig. 3). Among the top ten SBSsigns, one was unique for *RUNX1*::*RUNX1T1* (SBS88) and one for *CBFB*::*MYH11* (SBS16). Apart from SBS88, which has been linked to the genotoxic metabolite colibactin produced by *E. coli* and other enteric bacteria (https://cancer.sanger.ac.uk/signatures/sbs/sbs88/), the etiologies of the remaining top ten SBSsigns are unknown. Six of the common SBSsigns increased by age in a clock-like manner: SBS1, SBS5, SBS8, SBS19, SBS32, and SBS89 (Fig. 2).

The five most common IDsigns in the CBF AMLs were, in decreasing order, ID9, ID1, ID2, ID10, and ID5; the frequencies of these signatures did not differ significantly between *RUNX1*::*RUNX1T1*- and *CBFB*::*MYH11*-positive cases (Supplementary Fig. 4). ID9 has been correlated with mutations in *TP53*, genomic instability, and chromothripsis [11]. However, none of these features was present in our cases. ID1 and ID2 have been associated with slippage during DNA replication of the replicated (ID1) and template (ID2) strands (https://cancer.sanger.ac.uk/signatures/id/id1/, https://cancer.sanger.ac.uk/signatures/id/id2/), whereas the etiologies of ID5 and ID10 are unknown.

In conclusion, our findings suggest that the etiologies/mechanisms underlying transitions/transversions, SBSsigns, and IDsigns are similar in the two CBF AML types (Fig. 1 and Supplementary Figs. 1 and 4). Unfortunately, the etiologies of many of the common SBSsigns and IDsigns in the *RUNX1*::*RUNX1T1*- and *CBFB*::*MYH11*-positive cases are presently unknown. However, those with known or suspected origins can be dichotomized into i) spontaneous DNA changes/errors (SBS1, ID1, and ID2) and ii) associations with external agents, gene mutations, and ROS (SBS5, SBS18, SBS32, SBS88, and ID9). The lower frequency of SBS18 in adult *vs*. pediatric AML, despite being among the most common SBSsigns in the adult cases (Fig. 1 and Supplementary Fig. 3), may be explained by the fact that the SBS18 frequency did not increase with age in our patient cohort, whereas several other SBSsigns did (Fig. 2). In a recent study of pediatric AML, SBS18 was related to intrinsic ROS mechanisms that may have been induced already during fetal development [5]. Thus, if SBS18 occurs early on during the leukemogenic process of CBF AML, it would be more pronounced in childhood than in adult cases because the latter would have accumulated other age-related SBSsigns resulting in a relatively lower proportion of SBS18. Further studies of SBS18 and ROS-induced DNA damage in adult and childhood CBF AML are needed to clarify this issue.

## Supplementary information

Supplementary information

## Data Availability

The dataset generated during the current study will be made available in the EGA-SE depository upon its completion. Until then, the data are available from the corresponding author upon request through the following 10.17044/scilifelab.17082971 (WGS dataset). Supplementary information is available at Leukemia’s website.
