# Supplementary material for: Single base substitution and insertion/deletion mutational signatures in adult core binding factor acute myeloid leukemia
Source: Leukemia. 2022 Apr 1;36(6):1681–4. doi: 10.1038/s41375-022-01552-x (PMC9162923; doi:10.1038/s41375-022-01552-x)
Supplement: Supplementary file 1 — Supplementary information [file 41375_2022_1552_MOESM1_ESM.docx]

**Supplementary Information**

**Patients and whole genome sequencing**

Paired diagnostic and remission samples from 20 adults with core binding factor acute myeloid leukemia (AML), comprising ten with t(8;21)(q22;q22) [*RUNX1*::*RUNX1T1*] and ten with inv(16)(p13q22)/t(16;16)(p13;q22) [*CBFB*::*MYH11*], were analyzed by whole genome sequencing (Supplementary Table 1). The investigation was approved by the Research Ethics Committee of Lund University and informed consent for the analyses was obtained according to the Declaration of Helsinki.

DNA (2.5 µg) was extracted from diagnostic and remission bone marrow aspirates, after which sequencing libraries were constructed using the TruSeq PCR-Free DNA Library Preparation Kit (Illumina, San Diego, CA, USA) followed by cluster generation and 150 cycles paired-end sequencing with the NovaSeq 6000 system and v1.5 sequencing chemistry (Illumina) at the SNP&SEQ Technology Platform, Uppsala University. The paired-end reads were aligned to the human reference genome GRCh37 (hg19) by the Burrows-Wheeler Aligner tool (version 0.7.17) [1] and duplicate reads marking and local realignment were performed by GATK (version 4.0.11.0) [2].

**Analyses of SNV, DNV, indel, SV, UPID, CNA, and mutational signatures**

MuTect (version 1.1.7), Mutect2 (version 4.0.11.0), MuSE (version v1.0rc), and SvABA [2-5] were used to identify somatic single nucleotide variants (SNVs). Only SNVs with variant allele frequencies (VAFs) ≥0.2 and called by at least two methods were kept. Insertions/deletions (indels) up to 80 bp in size were identified using the cgpPindel pipeline [6] and those with VAFs ≥0.2 were retained for further analyses. The SNV and indel calling results were merged into a single vcf file for each sample and converted into a maf file by the vcf2maf package (https://github.com/mskcc/vcf2maf).

Somatic structural variants (SVs) were identified by GRIDSS using default settings [7] and Patchwork [8] was applied to ascertain uniparental isodisomies (UPIDs), *i.e.*, regions with copy number neutral loss of heterozygosity, and copy number abnormalities (CNAs; losses, gains, and chromothripsis).

The R package MutationalPatterns [9] was used to decompose mutational profiles into single base substitution mutational signatures (SBSsigns) (Wellcome Sanger Institute; v3.2 - March 2021; https://cancer.sanger.ac.uk/signatures/sbs/) and to ascertain the relative contributions of the different SBSsigns in each case. The indel signatures (IDsigns) were analyzed by the SigProfiler bioinformatic tool [10] and decomposed into mutational profiles based on pre-defined IDsigns (https://cancer.sanger.ac.uk/signatures/id).

Maftools [11] was used to determine the types and frequencies of all six transition/transversion types.

**Statistical tools**

Correlation coefficients between the number of transitions/transversions and the age of the patients were analyzed by Spearman's rank correlation coefficient and potential differences of the different transition/transversion types and mutational signatures between the *RUNX1*::*RUNX1T1*- and *CBFB*::*MYH11*-positive AML cases were analyzed with the Mann-Whitney U test. All statistical analyses were performed using R 4.1.1 with default setting (<https://www.r-project.org>). The G*Power software (version 3.1) [12] was used to determine the minimum required sample size for non-parametric statistical analysis based on Krzywinski et. al. [13].

**Data availability**

The dataset generated during the current study will be made available in the EGA-SE depository upon its completion. Until then, the data are available from the corresponding author upon request through the following DOI: https://doi.org/10.17044/scilifelab.17082971 (WGS dataset).

**REFERENCES**

1. Li H, Durbin R. Fast and accurate short read alignment with Burrows-Wheeler transform. Bioinformatics. 2009;25:1754-60.

2. DePristo MA, Banks E, Poplin R, Garimella KV, Maguire JR, Hartl C, et al. A framework for variation discovery and genotyping using next-generation DNA sequencing data. Nat Genet. 2011;43:491-8.

3. Cibulskis K, Lawrence MS, Carter SL, Sivachenko A, Jaffe D, Sougnez C, et al. Sensitive detection of somatic point mutations in impure and heterogeneous cancer samples. Nat Biotechnol. 2013;31:213-9.

4. Fan Y, Xi L, Hughes DST, Zhang J, Zhang J, Futreal PA, et al. MuSE: accounting for tumor heterogeneity using a sample-specific error model improves sensitivity and specificity in mutation calling from sequencing data. Genome Biol. 2016;17:178.

5. Wala JA, Bandopadhayay P, Greenwald NF, O'Rourke R, Sharpe T, Stewart C, et al. SvABA: genome-wide detection of structural variants and indels by local assembly. Genome Res. 2018;28:581-91.

6. Raine KM, Hinton J, Butler AP, Teague JW, Davies H, Tarpey P, et al. cgpPindel: identifying somatically acquired insertion and deletion events from paired end sequencing. Curr Protoc Bioinformatics. 2015;52:15.7.1-15.7.12.

7. Cameron DL, Schröder J, Penington JS, Do H, Molania R, Dobrovic A, et al. GRIDSS: sensitive and specific genomic rearrangement detection using positional de Bruijn graph assembly. Genome Research. 2017;27;2050-60.

8. Mayrhofer M, DiLorenzo S, Isaksson A. Patchwork: allele-specific copy number analysis of whole-genome sequenced tumor tissue. Genome Biol. 2013;14:R24.

9. Blokzijl F, Janssen R, van Boxtel R, Cuppen E. MutationalPatterns: comprehensive genome-wide analysis of mutational processes. Genome Med. 2018;10:33.

10. Bergstrom EN, Huang MN, Mahto U, Barnes M, Stratton MR, Rozen SG, et al. SigProfilerMatrixGenerator: a tool for visualizing and exploring patterns of small mutational events. BMC Genomics. 2019;20:685.

11. Mayakonda A, Lin D-C, Assenov Y, Plass C, Koeffler HP. Maftools: efficient and comprehensive analysis of somatic variants in cancer. Genome Res. 2018;28:1747-56.

12. Faul F, Erdfelder E, Lang AG, Buchner A. G*Power 3: a flexible statistical power analysis program for the social, behavioral, and biomedical sciences. Behav Res Methods. 2007;39:175-191.

13. Martin Krzywinski, Naomi Altman. Power and sample size. Nat Met. 2013;10:1139-40.

**Supplementary Table 1** Basic clinical and genetic data on the 20 adult core binding factor acute myeloid leukemias analyzed by whole genome sequencing

| Case | Sex | Age | Karyotype | Genomic breakpoints | Introns and genes targeted |
| --- | --- | --- | --- | --- | --- |
| No. |  | (years) |  | in the gene fusions^a^ |  |
|  |  |  |  |  |  |
| 1 | F | 30 | 46,XX,t(8;21)(q22;q22)[4]/46,XX[21] | Chr21:36212607::Chr8:93078842 | 6 (*RUNX1*) and 1 (*RUNX1T1*) |
| 2 | M | 32 | 45,X,-Y,t(8;21)(q22;q22)[25] | Chr21:36208480::Chr8:93082312 | 6 (*RUNX1*) and 1 (*RUNX1T1*) |
| 3 | M | 36 | 46,XY,t(8;21)(q22;q22)[16]/45,idem,-Y[9] | Chr21:36207095::Chr8:93083046 | 6 (*RUNX1*) and 1 (*RUNX1T1*) |
| 4^b^ | M | 52 | 46,XY,t(8;21)(q22;q22)[25] | Chr21:36228852::Chr8:93080853 | 6 (*RUNX1*) and 1 (*RUNX1T1*) |
| 5 | F | 53 | 45,XX,-7,t(8;21)(q22;q22)[25] | Chr21:36209361::Chr8:93120143 | 6 (*RUNX1*) and 1 (*RUNX1T1*) |
| 6 | F | 56 | 46,X,-X,+8,t(8;21)(q22;q22)[25] | Chr21:36221878::Chr8: 93079376 | 6 (*RUNX1*) and 1 (*RUNX1T1*) |
| 7 | F | 58 | 46,XX,t(8;21)(q22;q22)[25] | Chr21:36221372::Chr8:93080157 | 6 (*RUNX1*) and 1 (*RUNX1T1*) |
| 8 | F | 62 | 45,X,-X,t(8;21)(q22;q22)[25] | Chr21:36226551::Chr8:93077820 | 6 (*RUNX1*) and 1 (*RUNX1T1*) |
| 9 | M | 68 | 46,XY,t(8;21)(q22;q22)[25] | Chr21:36208182::Chr8:93081561 | 6 (*RUNX1*) and 1 (*RUNX1T1*) |
| 10 | M | 74 | 45,X,-Y,t(8;21)(q22;q22)[20]/46,XY[1] | Chr21:36221656::Chr8:93084066 | 6 (*RUNX1*) and 1 (*RUNX1T1*) |
| 11 | M | 19 | 47,XY,inv(16)(p13q22),+22[6] | Chr16:67129988::Chr16:15815005 | 5 (*CBFB*) and 33 (*MYH11*) |
| 12 | F | 23 | 46,XX,t(16;16)(p13;q22)[23]/46,XX[2] | Chr16:67127816::Chr16:15814928 | 5 (*CBFB*) and 33 (*MYH11*) |
| 13 | M | 23 | 46,XY,inv(16)(p13q22)[22]/48,idem,+4,+8[3] | Chr16:67121920::Chr16:15816461 | 5 (*CBFB*) and 33 (*MYH11*) |
| 14^b^ | F | 26 | 46,XX,inv(16)(p13q22)[25] | Chr16:67124790::Chr16:15815216 | 5 (*CBFB*) and 33 (*MYH11*) |
| 15 | F | 35 | 46,XX,t(16;16)(p13;q22)[25] | Chr16:67122846::Chr16:15814963 | 5 (*CBFB*) and 33 (*MYH11*) |
| 16^b^ | M | 44 | 46,XY,inv(16)(p13q22)[21]/46,XY[8] | Chr16:67131883::Chr16:15814915 | 5 (*CBFB*) and 33 (*MYH11*) |
| 17 | M | 49 | 46,XY,inv(16)(p13q22)[10]/46,XY[15] | Chr16:67120899::Chr16:15820476 | 5 (*CBFB*) and 33 (*MYH11*) |
| 18 | M | 54 | 46,XY,inv(16)(p13q22)[25] | Chr16:67129532::Chr16:15815307 | 5 (*CBFB*) and 33 (*MYH11*) |
| 19 | M | 55 | 48,XY,+8,inv(16)(p13q22),+22[23] | Chr16:67127575::Chr16:15815517 | 5 (*CBFB*) and 33 (*MYH11*) |
| 20 | M | 59 | 46,XY,inv(16)(p13q22)[13]/46,XY[11] | Chr16:67127796::Chr16:15814971 | 5 (*CBFB*) and 33 (*MYH11*) |

F, female; M, male.

^a^Breakpoint positions according to the human reference genome GRCh37 (hg19).

^b^The karyotypes of these cases have been previously published [1, 2].

**REFERENCES**

1. Billström R, Johansson B, Fioretos T, Garwicz S, Malm C, Zettervall O, et al. Poor survival in t(8;21)(q22;q22)-associated acute myeloid leukaemia with leukocytosis. Eur J Haematol. 1997;59:47-52.

2. Billström R, Ahlgren T, Békássy AN, Malm C, Olofsson T, Höglund M, et al. Acute myeloid leukemia with inv(16)(p13q22): involvement of cervical lymph nodes and tonsils is common and may be a negative prognostic sign. Am J Hematol. 2002;71:15-9.

**Supplementary Table 2** Genomic imbalances and uniparental isodisomies identified by whole genome sequencing of 20 adult core binding factor acute myeloid leukemias

| Case | Genetic | Chr.^a^ | Start^b^ | End^b^ | Size | Type of | Gene(s) present in abnormalities <1 Mb |
| --- | --- | --- | --- | --- | --- | --- | --- |
| No. | group |  |  |  | (Mb) | abnormality^c^ |  |
| 1 | t(8;21) | 17 | 71827696 | 72692020 | 0.86 | Gain | *AC103809.2*, *BTBD17*, *C17orf77, CD300A, CD300C*, *CD300E*, *CD300LB, CD300LD, CD300LF, DNAI2*, *GPRC5C, GPR142*, *KIF19, RAB37*, *RPL38*, *TTYH2* |
| 2 | t(8;21) | 7 | 30334033 | 30411116 | 0.08 | Loss | *ZNRF2* |
|  |  | 12 | 34359030 | 34560097 | 0.20 | UPID | No coding genes |
| 3 | t(8;21) | 12 | 7997028 | 8123767 | 0.13 | Gain | *SLC2A3*, *SLC2A14* |
|  |  | 16 | 2651804 | 2759456 | 0.11 | Gain | *KCTD5, PDPK1* |
| 4 | t(8;21) | 19 | 43244026 | 43362608 | 0.12 | Gain | *PSG3, PSG8* |
| 5 | t(8;21) | **7** | **0** | **159138663** | **159** | **Loss** |  |
|  |  | 8 | 42627883 | 43752615 | 1.12 | Gain |  |
| 6 | t(8;21) | 3 | 0 | 132655 | 0.13 | Gain | No coding genes |
|  |  | **8** | **0** | **146364022** | **146** | **Gain** |  |
|  |  | 9 | 136826183 | 136904825 | 0.08 | Gain | *BRD3*, *VAV2* |
|  |  | 10 | 135235945 | 135380555 | 0.14 | Gain | *AL161645.2*, *CYP2E1*, *SPRN, SYCE1* |
|  |  | 18 | 77638069 | 78077248 | 0.44 | UPID | *AC139100.2*, *ADNP2*, *HSBP1L1*, *KCNG2*, *PARD6G, PQLC1*, *RBFA*, |
|  |  |  |  |  |  |  | *RP11-795F19.5, TXNL4A* |
|  |  | **X** | **0** | **155270560** | **155** | **Loss** |  |
| 7 | t(8;21) | 5 | 32103895 | 32167210 | 0.06 | Gain | *GOLPH3*, *PDZD2* |
|  |  | 6 | 168332443 | 168597806 | 0.27 | Gain | *FRMD1*, *KIF25, MLLT4* |
|  |  | X | 7606191 | 8063138 | 0.46 | Gain | *PNPLA4, VCX* |
| 8 | t(8;21) | **X** | **0** | **155270560** | **155** | **Loss** |  |
| 9 | t(8;21) | 8 | 53416444 | 53855202 | 0.44 | Gain | *FAM150A, NPBWR1*, *RB1CC1* |
|  |  | 10 | 1932172 | 1988979 | 0.06 | Gain | No coding genes |
|  |  | 17 | 34436522 | 34490425 | 0.05 | Gain | No coding genes |
| 10 | t(8;21) | 1 | 161481733 | 161617663 | 0.14 | Gain | *FCGR2A*, *FCGR2B*, *FCGR3A*, *FCGR3B, HSPA6* |
|  |  | 15 | 30782770 | 31090985 | 0.31 | Gain | *ARHGAP11B, GOLGA8H*, *GOLGA8Q* |
|  |  | 16 | 21388796 | 21824955 | 0.44 | Gain | *IGSF6*, *METTL9*, *NPIPB3*, *OTOA* |
| 11 | inv(16) | 3 | 20007499 | 20116924 | 0.11 | Gain | *KAT2B, PP2D1, RAB5A* |
|  |  | 10 | 46528160 | 47965401 | 1.44 | Gain |  |
|  |  | **22** | **0** | **51304566** | **51** | **Gain** |  |
| 12 | inv(16) | 17 | 29009895 | 30367180 | 1.36 | Loss |  |
| 13 | inv(16) | 5 | 120120862 | 120859756 | 0.74 | Gain | No coding genes |
| 15 | inv(16) | 5 | 100716157 | 100775597 | 0.06 | Loss | No coding genes |
|  |  | 7 | 3087076 | 3179981 | 0.09 | Gain | *AC091801.1* |
|  |  | 11 | 82052805 | 82110174 | 0.06 | Loss | No coding genes |
|  |  | 19 | 42733855 | 59128983 | 16 | UPID |  |
| 16 | inv(16) | 16 | 15814707 | 16292118 | 0.48 | Loss | *ABCC1*, *ABCC6, FOPNL*, *MYH11, NDE1* |
|  |  | 17 | 34436728 | 34490425 | 0.05 | Gain | No coding genes |
| 17 | inv(16) | 5 | 105833546 | 116333541 | 10 | Loss |  |
|  |  | 15 | 0 | 75533185 | 75 | UPID |  |
|  |  | 15 | 76657864 | 102531392 | 26 | UPID |  |
| 18 |  | 19 | 54739140 | 54847961 | 0.11 | Gain | *LILRA3*, *LILRA4, LILRA5, LILRA6*, *LILRB2*, *LILRB3*, *LILRB5*, *RPS9* |
| 19 | inv(16) | **8** | **0** | **146364022** | **146** | **Gain** |  |
|  |  | **22** | **0** | **51304566** | **51** | **Gain** |  |
| 20 | inv(16) | 3 | 35800757 | 35945617 | 0.14 | Gain | *ARPP21* |
|  |  | 12 | 63934745 | 64129264 | 0.19 | Gain | *DPY19L2* |
|  |  | 16 | 75164965 | 75428186 | 0.26 | Gain | *BCAR1, CFDP1, CTRB1*, *CTRB2*, *ZFP1* |

Chr, chromosome; UPID, uniparental isodisomy.

^a^The Y chromosome was not included in the analyses.

^b^Breakpoint positions according to the human reference genome GRCh37 (hg19).

^c^Imbalances in bold type were also identified by the conventional G-banding analysis.

**Supplementary Table 3** Nonsynonymous variants in coding genes identified by whole genome sequencing of 20 adult core binding factor acute myeloid leukemias

| Gene^a^ | Case | Variant^b^ | Type of | Genomic | VAF | SIFT^d^ | PolyPhen^d^ | dbSNPrs |
| --- | --- | --- | --- | --- | --- | --- | --- | --- |
|  | No. |  | change | position^c^ | (%) |  |  |  |
| *A2M* | 4 | c.229G>T | Missense | 12:9265997 | 50 | Tolerated | Benign | No data |
| *ABCA3* | 6 | c.1616A>C | Missense | 16:2349529 | 59 | Deleterious | Probably damaging | Novel |
| *ABCA13* | 14 | c.3029C>A | Missense | 7:48312292 | 56 | Deleterious | Possibly damaging | rs377050338 |
| *ABCC10* | 10 | c.3761C>A | Missense | 6:43415477 | 57 | Tolerated | Benign | Novel |
| *AC002472.1* | 15 | c.163C>T | Missense | 22:21360574 | 48 | Deleterious | Probably damaging | Novel |
| *ACAN* | 7 | c.1189G>A | Missense | 15:89388873 | 37 | Deleterious | Probably damaging | rs748422617 |
|  |  |  |  |  |  | (low confidence) |  |  |
| *ADAM18* | 8 | c.92G>A | Missense | 8:39442831 | 34 | Tolerated | Benign | rs374874930 |
| *ADRM1* | 13 | c.728-735delGCCCCGCG | Frameshift | 20:60882756- | 44 | Not scored | Not scored | Novel |
|  |  |  | deletion | 60882763 |  |  |  |  |
| *AHCTF1* | 16 | c.4534A>C | Missense | 1:247016449 | 51 | Deleterious | Benign | Novel |
| *AK7* | 19 | c.1742C>T | Missense | 14:96944988 | 26 | Deleterious | Probably damaging | rs201624359 |
| *AKAP9* | 7 | c.11680-11681delAC | Frameshift | 7:91737940- | 45 | Not scored | Not scored | Novel |
|  |  |  | deletion | 91737941 |  |  |  |  |
| *AMOT* | 20 | c.687G>C | Missense | X:112065668 | 95 | Deleterious | Benign | Novel |
| *APOB* | 5 | c.11333G>A | Missense | 2:21228407 | 42 | Deleterious | Benign | Novel |
| *ARID1A* | 15 | c.833G>A | Missense | 1:27023727 | 53 | Deleterious | Benign | No data |
|  |  |  |  |  |  | (low confidence) |  |  |
| *ASPM* | 16 | c.5084C>T | Missense | 1:197073297 | 34 | Tolerated | Benign | rs541097917 |
| ***ASXL2*** | 3 | c.2219-2222insCTCG | Frameshift | 2:25966983- | 33 | Not scored | Not scored | Novel |
|  |  |  | insertion | 25966984 |  |  |  |  |
| ***ASXL2*** | 7 | c.2351-2352insGG | Frameshift | 2:25966853- | 28 | Not scored | Not scored | Novel |
|  |  |  | insertion | 25966854 |  |  |  |  |
| *ATP1A2* | 13 | c.682G>A | Missense | 1:160094977 | 54 | Deleterious | Benign | rs1218350015 |
| *BAI2* | 20 | c.4237C>T | Missense | 1:32196544 | 33 | Tolerated | Benign | Novel |
| *BASP1* | 7 | c.379G>A | Missense | 5:17275704 | 44 | Deleterious | Benign | rs1442362061 |
| *C1orf173* | 8 | c.2585G>A | Missense | 1:75038809 | 58 | Tolerated | Benign | Novel |
| *C6orf25* | 17 | c.59G>C | Missense | 6:31691219 | 40 | Tolerated | Benign | Novel |
| *C9orf72* | 4 | c.451G>A | Nonsense | 9:27565582 | 52 | Unknown | Unknown | Novel |
| *C11orf68* | 9 | c.231G>T | Missense | 11:65685581 | 50 | Deleterious | Benign | Novel |
| *CACNA2D2* | 9 | c.3242T>G | Missense | 3:50402389 | 41 | Deleterious | Benign | Novel |
| *CAMSAP3* | 17 | c.1661C>T | Missense | 19:7676959 | 25 | Tolerated | Benign | rs200164598 |
| *CATSPER2* | 9 | c.1558C>T | Missense | 15:43924400 | 51 | Deleterious | Possibly damaging | Novel |
| *CCDC88B* | 12 | c.1846G>A | Missense | 11:64111859 | 51 | Tolerated | Benign | Novel |
| *CCND2* | 7 | c.841C>A | Missense | 12:4409146 | 40 | Deleterious | Probably damaging | No data |
| *CD101* | 18 | c.196C>T | Missense | 1:117552624 | 53 | Deleterious | Probably damaging | No data |
| *CDH11* | 13 | c.1551G>T | Missense | 16:65005573 | 53 | Deleterious | Possibly damaging | Novel |
| *CEP164* | 9 | c.1175T>G | Missense | 11:117244489 | 40 | Deleterious | Benign | Novel |
| *CFTR* | 6 | c.4282C>A | Missense | 7:117307001 | 51 | Tolerated | Probably damaging | Novel |
| ***CNTN5*** | 2 | c.2995C>T | Missense | 11:100211902 | 49 | Tolerated | Benign | Novel |
| ***CNTN5*** | 4 | c.2755C>G | Missense | 11:100211219 | 50 | Tolerated | Benign | Novel |
| *COL5A2* | 18 | c.2968C>T | Missense | 2:189915366 | 46 | Deleterious | Probably damaging | rs1040238147 |
| *COL16A1* | 10 | c.1286C>T | Missense | 1:32157215 | 62 | Deleterious | Probably damaging | Novel |
| *COL22A1* | 7 | c.4565C>T | Missense | 8:139606310 | 41 | Tolerated | Benign | rs747819697 |
| *COQ10B* | 10 | c.20A>G | Missense | 2:198318304 | 48 | Tolerated | Benign | rs752270714 |
| *CREBBP* | 3 | c.5450G>T | Missense | 16:3779598 | 26 | Deleterious | Probably damaging | No data |
| ***CSF3R*** | 4 | c.1853G>A | Missense | 1:36933434 | 58 | Deleterious | Probably damaging | rs796065343 |
| ***CSF3R*** | 9 | c.2427insG | Frameshift | 1:36932122- | 11 | Not scored | Not scored | Novel |
|  |  |  | insertion | 36932123 |  |  |  |  |
| ***CSF3R*** | 11 | c.2427insCTCG | Frameshift | 1:36932122- | 23 | Not scored | Not scored | Novel |
|  |  |  | insertion | 3693212 |  |  |  |  |
| *CSRP3* | 15 | c.159C>A | Missense | 11:19209805 | 30 | Tolerated | Benign | Novel |
| *CT55* | 16 | c.295G>A | Missense | X:134294465 | 86 | Deleterious | Benign | rs199770128 |
| *CXCR6* | 2 | c.802T>C | Missense | 3:45988775 | 36 | Tolerated | Probably damaging | Novel |
| *CYP2J2* | 8 | c.1066G>A | Missense | 1:60370668 | 32 | Deleterious | Probably damaging | rs201789916 |
| *DDX51* | 10 | c.1514G>A | Missense | 12:132625207 | 34 | Deleterious | Benign | rs764746864 |
| *DDX60* | 6 | c.3892C>T | Missense | 4:169169410 | 48 | Tolerated | Benign | Novel |
| *DHX29* | 20 | c.1906C>T | Missense | 5:54579090 | 42 | Tolerated | Benign | Novel |
| *DLG5* | 6 | c.4945C>T | Missense | 10:79566538 | 36 | Deleterious | Probably damaging | rs1474755701 |
| *DMRT1* | 17 | c.838A>C | Missense | 9:916778 | 22 | Deleterious | Benign | Novel |
| *DNAH9* | 5 | c.3239T>G | Missense | 17:11572997 | 50 | Deleterious | Possibly damaging | Novel |
| *ECM1* | 9 | c.863G>A | Missense | 1:150484006 | 45 | Tolerated | Benign | rs138083168 |
| *EML4* | 4 | c.1068delT | Frameshift | 2:42513464 | 52 | Not scored | Not scored | Novel |
|  |  |  | deletion |  |  |  |  |  |
| *EPPK1* | 15 | c.2402G>A | Missense | 8:144945020 | 25 | Tolerated | Benign | No data |
| *FAM47C* | 13 | c.2384G>A | Missense | X:37028867 | 90 | Tolerated | Possibly damaging | No data |
| *FAM83A* | 8 | c.91G>T | Missense | 8:124195187 | 45 | Tolerated | Benign | Novel |
| *FAM155B* | 14 | c.28G>A | Missense | X:68725153 | 39 | Tolerated | Benign | rs774340761 |
| *FBN2* | 15 | c.7879C>T | Missense | 5:127607772 | 22 | Deleterious | Possibly damaging | rs369568420 |
| *FBN3* | 9 | c.1099C>T | Missense | 19:8203127 | 46 | Tolerated | Benign | rs766315700 |
| *FBXL18* | 14 | c.89C>G | Missense | 7:5545191 | 50 | Tolerated | Benign | rs113643930 |
| *FCHO1* | 2 | c.2442-2443insTGGCTC | Frameshift | 19:17897398- | 26 | Not scored | Not scored | Novel |
|  |  | GTTGGC | insertion | 17897399 |  |  |  |  |
| *FLG* | 8 | c.2105G>T | Nonsense | 1:152285257 | 26 | Unknown | Unknown | No data |
| ***FLT3*** | 6 | c.1734-1769insAAG | Frameshift | 13:28608286- | 17 | Not scored | Not scored | Novel |
|  |  | TACTCATTATCT | insertion | 28608287 |  |  |  |  |
|  |  | GAGGAGCCGGT |  |  |  |  |  |  |
|  |  | CACCTGTACC |  |  |  |  |  |  |
| ***FLT3*** | 8 | c.1784-1785insCTGAAAT | Frameshift | 13:28608271- | 17 | Not scored | Not scored | Novel |
|  |  | CAACGTAGAAGTACTC | insertion | 28608272 |  |  |  |  |
|  |  | ATTATCTGAGGAGCC |  |  |  |  |  |  |
|  |  | GGGA |  |  |  |  |  |  |
| ***FLT3*** | 15 | c.2812-2820dup | Frameshift | 13:28588627- | 22 | Not scored | Not scored | Novel |
|  |  | AGTCAAATT | insertion | 28588628 |  |  |  |  |
| ***FLT3*** | 15 | c.2039G>A | Missense | 13:28602329 | 25 | Deleterious | Probably damaging | rs372303125 |
| ***FLT3*** | 17 | c.2516T>C | Missense | 13:28592629 | 23 | Deleterious | Possibly damaging | rs991132188 |
| *FN1* | 3 | c.2305G>A | Missense | 2:216273144 | 49 | Deleterious | Probably damaging | No data |
| *FUS* | 15 | c.439C>T | Nonsense | 16:31195633 | 49 | Unknown | Unknown | Novel |
| *GALNT6* | 8 | c.1505T>C | Missense | 12:51751230 | 45 | Tolerated | Benign | Novel |
| *GDF6* | 2 | c.488C>T | Missense | 8:97157671 | 29 | Deleterious | Probably damaging | rs1244838008 |
| *GFAP* | 16 | c.230T>C | Missense | 17:42992625 | 46 | Deleterious | Probably damaging | rs57590980 |
| *GJC3* | 8 | c.626T>C | Missense | 7:99526618 | 47 | Deleterious | Probably damaging | Novel |
| *GLI3* | 16 | c.3823C>T | Missense | 7:42004848 | 41 | Tolerated | Benign | rs756156901 |
| *GOLGA6L2* | 17 | c.32G>A | Missense | 15:23692297 | 51 | Deleterious | Probably damaging | Novel |
|  |  |  |  |  |  | (low confidence) |  |  |
| *GPR17* | 12 | c.923G>A | Missense | 2:128409148 | 46 | Deleterious | Probably damaging | rs554870046 |
| *GPR56* | 12 | c.1774-1803delCCATGG | In-frame | 16:57695698- | 56 | Not scored | Not scored | Novel |
|  |  | TGGTGCAGATCCTG | deletion | 57695727 |  |  |  |  |
|  |  | CGGCTGCGCC |  |  |  |  |  |  |
| *HABP4* | 8 | c.652delA | Frameshift | 9:99227756 | 29 | Not scored | Not scored | Novel |
|  |  |  | deletion |  |  |  |  |  |
| *HCFC1* | 4 | c.164A>G | Missense | X:153236128 | 95 | Deleterious | Probably damaging | Novel |
| *HEATR5A* | 5 | c.2254C>T | Missense | 14:31828000 | 42 | Tolerated | Benign | Novel |
| *HNRNPH3* | 10 | c.806C>G | Missense | 10:70101372 | 34 | Deleterious | Probably damaging | Novel |
| *HYAL1* | 19 | c.673T>C | Missense | 3:50339715 | 32 | Tolerated | Benign | Novel |
| *HYAL3* | 8 | c.383C>T | Nonsense | 3:50332651 | 35 | Unknown | Unknown | No data |
| *HYDIN* | 10 | c.9319C>T | Missense | 16:70926362 | 33 | Deleterious | Probably damaging | No data |
| *IAH1* | 7 | c.244G>C | Missense | 2:9618460 | 36 | Deleterious | Possibly damaging | No data |
| *IDH2* | 5 | c.419C>T | Missense | 15:90631934 | 43 | Deleterious | Probably damaging | rs121913502 |
|  |  |  |  |  |  | (low confidence) |  |  |
| *IGHV3-20* | 9 | c.46C>A | Missense | 14:106667988 | 54 | Deleterious | Possibly damaging | Novel |
|  |  |  |  |  |  | (low confidence) |  |  |
| *IGKV3D-20* | 18 | c.130G>A | Missense | 2:90078093 | 53 | Tolerated | Benign | rs2555982 |
| *IGSF21* | 9 | c.487G>T | Missense | 1:18688671 | 44 | Tolerated | Benign | Novel |
| *IL20RA* | 4 | c.578G>A | Missense | 6:137330455 | 40 | Tolerated | Benign | rs142288648 |
| *INADL* | 19 | c.2362A>G | Missense | 1:62327262 | 25 | Deleterious | Benign | Novel |
| *INADL* | 19 | c.2364C>G | Missense | 1:62327264 | 25 | Tolerated | Benign | Novel |
| *ING1* | 18 | c.163A>G | Missense | 13:111367953 | 41 | Tolerated | Benign | Novel |
| *INTS5* | 11 | c.1726G>A | Missense | 11:62415826 | 42 | Tolerated | Benign | rs201064662 |
| *JAM2* | 10 | c.809A>C | Missense | 21:27081749 | 46 | Tolerated | Benign | Novel |
| *JUP* | 10 | c.1659insA | Frameshift | 17:39914764- | 49 | Not scored | Not scored | Novel |
|  |  |  | insertion | 39914765 |  |  |  |  |
| *KCNG2* | 3 | c.125G>A | Missense | 18:77623792 | 33 | Deleterious | Probably damaging | rs749338394 |
| *KCNK10* | 17 | c.139G>A | Missense | 14:88729809 | 47 | Deleterious | Probably damaging | rs138379553 |
|  |  |  |  |  |  | (low confidence) |  |  |
| *KCNQ3* | 13 | c.860G>A | Missense | 8:133187773 | 54 | Tolerated | Benign | rs531151809 |
| *KIAA2022* | 4 | c.2432G>T | Missense | X:73961960 | 100 | Del | Probably damaging | rs750889767 |
| *KIF2B* | 17 | c.520C>T | Missense | 17:51900914 | 42 | Del | Probably damaging | rs769176818 |
| ***KIT*** | 2 | c.2458G>T | Missense | 4:55599332 | 27 | Deleterious | Probably damaging | rs1057519710 |
| ***KIT*** | 3 | c.1255-1257delACG | Frameshift | 4:55589771- | 17 | Not scored | Not scored | Novel |
| ***KIT*** | 5 | c.2447A>T | Missense | 4:55599321 | 26 | Deleterious | Probably damaging | rs121913507 |
| ***KIT*** | 13 | c.2446G>T | Missense | 4:55599320 | 45 | Deleterious | Probably damaging | rs121913506 |
| ***KIT*** | 18 | c.2447A>T | Missense | 4:55599321 | 49 | Deleterious | Probably damaging | rs121913507 |
| ***KIT*** | 19 | c.2447A>T | Missense | 4:55599321 | 29 | Deleterious | Probably damaging | rs121913507 |
| ***KRAS*** | 14 | c.182T>A | Missense | 12:25380276 | 44 | Deleterious | Possibly damaging | rs121913240 |
| ***KRAS*** | 16 | c.38C>T | Missense | 12:25398281 | 22 | Deleterious | Benign | rs112445441 |
| *LHX3* | 4 | c.626C>T | Nonsense | 9:139090662 | 49 | Unknown | Unknown | Novel |
| *LIFR* | 4 | c.1840C>T | Missense | 5:38496529 | 52 | Deleterious | Probably damaging | Novel |
| *LIMK2* | 19 | c.457T>G | Missense | 22:31656032 | 47 | Tolerated | Benign | Novel |
| *LRIT2* | 10 | c.130C>G | Missense | 10:85984851 | 38 | Tolerated | Benign | rs545034368 |
| *LRP1B* | 3 | c.10790C>T | Missense | 2:141128833 | 62 | Tolerated | Benign | rs752964745 |
| *LRP2* | 8 | c.3313C>T | Missense | 2:170101320 | 48 | Tolerated | Benign | rs377021833 |
| *LRRC17* | 10 | c.938delT | Frameshift | 7:102584661 | 46 | Not scored | Not scored | No data |
|  |  |  | deletion |  |  |  |  |  |
| *LRRFIP1* | 9 | c.1543A>G | Missense | 2:238671899 | 48 | Tolerated | Benign | rs745926152 |
| *MAP3K13* | 20 | c.2399C>T | Missense | 3:185191518 | 30 | Tolerated | Benign | rs767698480 |
| *MEOX2* | 18 | c.912T>A | Missense | 7:15652015 | 46 | Deleterious | Probably damaging | Novel |
|  |  |  |  |  |  | (low confidence) |  |  |
| *MFSD1* | 17 | c.383C>T | Missense | 3:158523170 | 52 | Deleterious | Possibly damaging | rs140699673 |
| *MIB2* | 9 | c.1073C>T | Missense | 1:1560672 | 47 | Tolerated | Benign | rs755009511 |
| *MKI67* | 11 | c.5284C>T | Missense | 10:129904820 | 41 | Tolerated | Possibly damaging | rs368269373 |
| *MROH5* | 7 | c.44G>A | Missense | 8:142517206 | 29 | Tolerated | Benign | rs79802566 |
| *MTA2* | 9 | c.878T>C | Missense | 11:62364113 | 57 | Deleterious | Probably damaging | rs777871681 |
| *MYH3* | 12 | c.4282C>T | Missense | 17:10538231 | 45 | Deleterious | Probably damaging | No data |
| *MYH8* | 6 | c.4196C>T | Missense | 17:10300286 | 47 | Deleterious | Benign | rs368200953 |
| *MYO18B* | 10 | c.244G>A | Missense | 22:26164127 | 25 | Tolerated | Benign | Novel |
| *MYOZ1* | 8 | c.427-428insCACAG | Frameshift | 10:75394316- | 33 | Not scored | Not scored | Novel |
|  |  |  | insertion | 75394317 |  |  |  |  |
| *MYT1L* | 2 | c.2341G>A | Missense | 2:1893186 | 42 | Deleterious | Possibly damaging | rs756426615 |
| *NBEAL2* | 4 | c.2257G>T | Missense | 3:47037866 | 46 | Tolerated | Benign | rs780140037 |
| *NBPF9* | 7 | c.1876C>A | Missense | 1:144828609 | 42 | Tolerated | Possibly damaging | Novel |
| *NF1* | 20 | c.6073T>C | Missense | 17:29663417 | 35 | Deleterious | Probably damaging | Novel |
| *NISCH* | 18 | c.4064A>G | Missense | 3:52526047 | 47 | Tolerated | Benign | Novel |
| *NKPD1* | 4 | c.436C>T | Missense | 19:45662014 | 46 | Tolerated | Benign | rs1191549016 |
| *NOC2L* | 5 | c.1868C>T | Missense | 1:881602 | 42 | Deleterious | Benign | rs148962722 |
| *NPTXR* | 19 | c.544-545insCGAA | Frameshift | 22:39239319- | 12 | Not scored | Not scored | Novel |
|  |  |  | insertion | 39239320 |  |  |  |  |
| *NUP107* | 2 | c.938T>C | Missense | 12:69107557 | 47 | Tolerated | Benign | rs1198848766 |
| *NUP188* | 19 | c.4771G>T | Missense | 9:131767957 | 31 | Tolerated | Benign | Novel |
| *OPN5* | 10 | c.360C>A | Missense | 6:47759647 | 62 | Deleterious | Probably damaging | No data |
| *OR5P2* | 20 | c.325C>T | Missense | 11:7818165 | 36 | Deleterious | Benign | rs748657480 |
| *OR6B3* | 8 | c.807G>T | Missense | 2:240984683 | 46 | Deleterious | Possibly damaging | Novel |
| *OR51G1* | 3 | c.409C>T | Missense | 11:4945161 | 50 | Tolerated | Benign | rs145607331 |
| *ORC1* | 10 | c.871T>G | Missense | 1:52859326 | 49 | Tolerated | Benign | rs552278270 |
| *OTUD5* | 5 | c.442G>A | Missense | X:48814391 | 40 | Tolerated | Benign | Novel |
| *PARM1* | 3 | c.362C>T | Missense | 4:75937953 | 48 | Tolerated | Benign | rs963350600 |
| *PBX3* | 5 | c.364G>C | Missense | 9:128678054 | 47 | Tolerated | Probably damaging | rs1207671653 |
| *PCDHA6* | 10 | c.796G>T | Missense | 5:140208472 | 41 | Deleterious | Probably damaging | Novel |
|  |  |  |  |  |  | (low confidence) |  |  |
| *PCDHA12* | 5 | c.557A>C | Missense | 5:140255614 | 54 | Tolerated | Benign | Novel |
| *PEX26* | 2 | c.890-891insGA | Frameshift | 22:18570812- | 10 | Not scored | Not scored | Novel |
|  |  |  | insertion | 18570813 |  |  |  |  |
| *PLCH2* | 11 | c.2947C>G | Missense | 1:2433917 | 33 | Tolerated | Benign | Novel |
| *PMVK* | 20 | c.79C>A | Nonsense | 1:154909083 | 48 | Unknown | Unknown | rs373000976 |
| *PNPLA3* | 2 | c.1069C>T | Missense | 22:44335962 | 56 | Deleterious | Possibly damaging | rs199525362 |
| *PNPLA6* | 20 | c.2548C>T | Missense | 19:7619493 | 42 | Deleterious | Possibly damaging | Novel |
| *PODN* | 16 | c.217G>A | Missense | 1:53535600 | 36 | Tolerated | Benign | rs1278850787 |
| *PPFIBP2* | 4 | c.881A>G | Missense | 11:7649570 | 44 | Tolerated | Possibly damaging | Novel |
| *PPP2R1B* | 20 | c.1327C>T | Missense | 11:111622894 | 27 | Deleterious | Probably damaging | Novel |
| *PRAMEF10* | 5 | c.927-928TG>AT | Missense | 1:12953244- | 45 | Unknown | Unknown | Novel |
|  |  |  |  | 12953245 |  |  |  |  |
| *PROKR2* | 8 | c.889C>T | Missense | 20:5282952 | 44 | Tolerated | Possibly damaging | rs139399061 |
| *PTPN11* | 11 | c.794G>A | Missense | 12:112910785 | 33 | Deleterious | Possibly damaging | rs376607329 |
| *PYCR2* | 14 | c.303G>C | Missense | 1:226109919 | 58 | Deleterious | Probably damaging | Novel |
| *RAB3GAP2* | 10 | c.1900-1901delTG | Frameshift | 1:220357475- | 42 | Not scored | Not scored | Novel |
|  |  |  | deletion | 220357476 |  |  |  |  |
| *RAD21* | 6 | c.1497insT | Frameshift | 8:117862979- | 48 | Not scored | Not scored | Novel |
|  |  |  | deletion | 117862980 |  |  |  |  |
| *RBL1* | 20 | c.2758T>C | Missense | 20:35635927 | 45 | Deleterious | Benign | Novel |
| *RBM6* | 7 | c.2020G>C | Missense | 3:50095885 | 45 | Tolerated | Possibly damaging | Novel |
| *RGSL1* | 6 | c.1431G>C | Missense | 1:182443677 | 34 | Deleterious | Possibly damaging | Novel |
| *RHPN2* | 4 | c.713G>A | Missense | 19:33498967 | 31 | Tolerated | Benign | rs142413151 |
| *SEMA5B* | 10 | c.605C>A | Missense | 3:122658303 | 54 | Deleterious | Probably damaging | Novel |
| *SERPINA10* | 3 | c.31GC>T | Missense | 14:94756900 | 32 | Tolerated | Benign | rs759069270 |
| *SERPINB5* | 5 | c.272G>A | Missense | 18:61154282 | 35 | Tolerated | Benign | rs146056603 |
| *SETBP1* | 12 | c.2947C>G | Missense | 18:42532252 | 43 | Deleterious | Probably damaging | No data |
| *SGK2* | 6 | c.286G>A | Missense | 20:42196324 | 36 | Tolerated | Benign | rs200530019 |
| *SH2D3A* | 20 | c.1669G>A | Missense | 19:6752666 | 23 | Deleterious | Benign | rs910175229 |
| *SHANK2* | 7 | c.112G>A | Missense | 11:70858261 | 43 | Deleterious | Probably damaging | rs369954613 |
| *SHISA6* | 4 | c.1192C>T | Nonsense | 17:11461157 | 51 | Unknown | Unknown | Novel |
| *SLC25A3* | 20 | c.143C>T | Missense | 12:98987899 | 47 | Deleterious | Benign | Novel |
| *SLC51B* | 10 | c.113A>G | Missense | 15:65343868 | 44 | Tolerated | Benign | Novel |
| *SLITRK3* | 7 | c.1724T>G | Missense | 3:164906895 | 32 | Tolerated | Benign | Novel |
| *SMARCA2* | 4 | c.4009A>G | Missense | 9:2161713 | 48 | Tolerated | Benign | Novel |
| *SMC3* | 7 | c.1983-1985insGGG | In-frame | 10:112356173- | 51 | Not scored | Not scored | Novel |
|  |  |  | insertion | 112356174 |  |  |  |  |
| *SND1* | 5 | c.1207C>T | Missense | 7:127447592 | 80 | Deleterious | Probably damaging | rs771547339 |
| *SPG7* | 6 | c.458G>C | Missense | 16:89590495 | 42 | Tolerated | Possibly damaging | Novel |
| *SPICE1* | 17 | c.1789T>C | Missense | 3:113172666 | 26 | Deleterious | Benign | Novel |
| *STAT3* | 4 | c.1855C>A | Missense | 17:40475055 | 27 | Deleterious | Benign | Novel |
| *SULT1E1* | 19 | c.416T>C | Missense | 4:70715235 | 24 | Deleterious | Probably damaging | Novel |
| *SUMF1* | 4 | c.1106C>T | Missense | 3:4403847 | 40 | Tolerated | Benign | rs926687493 |
| *SYNE1* | 6 | c.15562C>T | Missense | 6:152646314 | 40 | Unknown | Benign | rs942243786 |
| *SYT12* | 12 | c.1154G>A | Missense | 11:66816116 | 48 | Deleterious | Probably damaging | Novel |
| *TERT* | 8 | c.2843C>T | Missense | 5:1264519 | 47 | Tolerated | Possibly damaging | Novel |
| *TMEM130* | 16 | c.86C>T | Missense | 7:98461023 | 42 | Tolerated | Benign | Novel |
| *TMEM184C* | 18 | c.407G>A | Missense | 4:148546070 | 50 | Tolerated | Benign | rs758084153 |
| *TMEM230* | 7 | c.497G>A | Missense | 20:5081492 | 34 | Deleterious | Possibly damaging | rs1405909337 |
| *TRIO* | 15 | c.298C>T | Nonsense | 5:14280496 | 40 | Unknown | Unknown | No data |
| *TRRAP* | 18 | c.4873C>T | Missense | 7:98547145 | 63 | Deleterious | Benign | rs150375708 |
| *TTC3* | 8 | c.3604-3605insCTC | In-frame | 21:38538119- | 32 | Not scored | Not scored | Novel |
|  |  |  | insertion | 38538120 |  |  |  |  |
| *UNC45B* | 17 | c.1055GG>T | Missense | 17:33491089 | 24 | Tolerated | Possibly damaging | rs776382433 |
| *USH2A* | 18 | c.6047CG>C | Missense | 1:216243445 | 53 | Deleterious | Benign | rs750260175 |
| *USP10* | 8 | c.1277TT>G | Missense | 16:84792406 | 43 | Deleterious | Probably damaging | Novel |
| *WDR27* | 19 | c.17AT>A | Missense | 6:170089085 | 35 | Deleterious | Benign | rs771666317 |
| *WDR76* | 9 | c.1477G>T | Nonsense | 15:44150936 | 62 | Unknown | Unknown | No data |
| *WT1* | 17 | c.534delC | Frameshift | 11:32456358 | 23 | Not scored | Not scored | Novel |
|  |  |  | deletion |  |  |  |  |  |
| ***ZBTB7A*** | 8 | c.254A>C | Missense | 19:4054977 | 50 | Deleterious | Probably damaging | Novel |
| ***ZBTB7A*** | 10 | c.1195G>A | Nonsense | 19:4054036 | 55 | Unknown | Unknown | No data |
| *ZC3H3* | 8 | c.1199C>T | Missense | 8:144620338 | 51 | Tolerated | Benign | Novel |
| *ZFHX4* | 18 | c.5944G>T | Missense | 8:77765101 | 51 | Tolerated | Probably damaging | Novel |
| *ZFPM2* | 4 | c.1840G>C | Missense | 8:106814150 | 45 | Tolerated | Benign | Novel |
| *ZNF217* | 13 | c.724insT | Frameshift | 20:52198641- | 50 | Not scored | Not scored | Novel |
|  |  |  | insertion | 52198642 |  |  |  |  |
| *ZNF407* | 16 | c.3657A>G | Missense | 18:72346632 | 39 | Tolerated | Benign | rs1360307687 |
| *ZNF646* | 5 | c.1412-1415delGGTC | Frameshift | 16:31089056- | 41 | Not scored | Not scored | Novel |
|  |  |  | deletion | 31089059 |  |  |  |  |
| *ZSCAN21* | 6 | c.956A>C | Missense | 7:99661774 | 47 | Tolerated | Benign | Novel |

dbSNPrs, single nucleotide polymorphism database reference number; VAF, variant allele frequency.

^a^Genes targeted in more than one case are indicated in bold type.

^b^Variants are described according to the HGVS nomenclature (https://www.hgvs.org/mutnomen/).

^c^Positions according to the human reference genome GRCh37 (hg19).

^d^The predictions provided by SIFT and PolyPhen are based on probability scores. In SIFT, a variant is predicted to be damaging if the score is ≤0.05 and tolerated if the score is >0.05. In PolyPhen, variants with scores between 0.0 and 0.045 are predicted to be benign, whereas scores from 0.045 to 1.0 are increasingly confidently predicted to be deleterious. Insertions and deletions are not scored by SIFT or PolyPhen.

**Supplementary Figure 1**

**
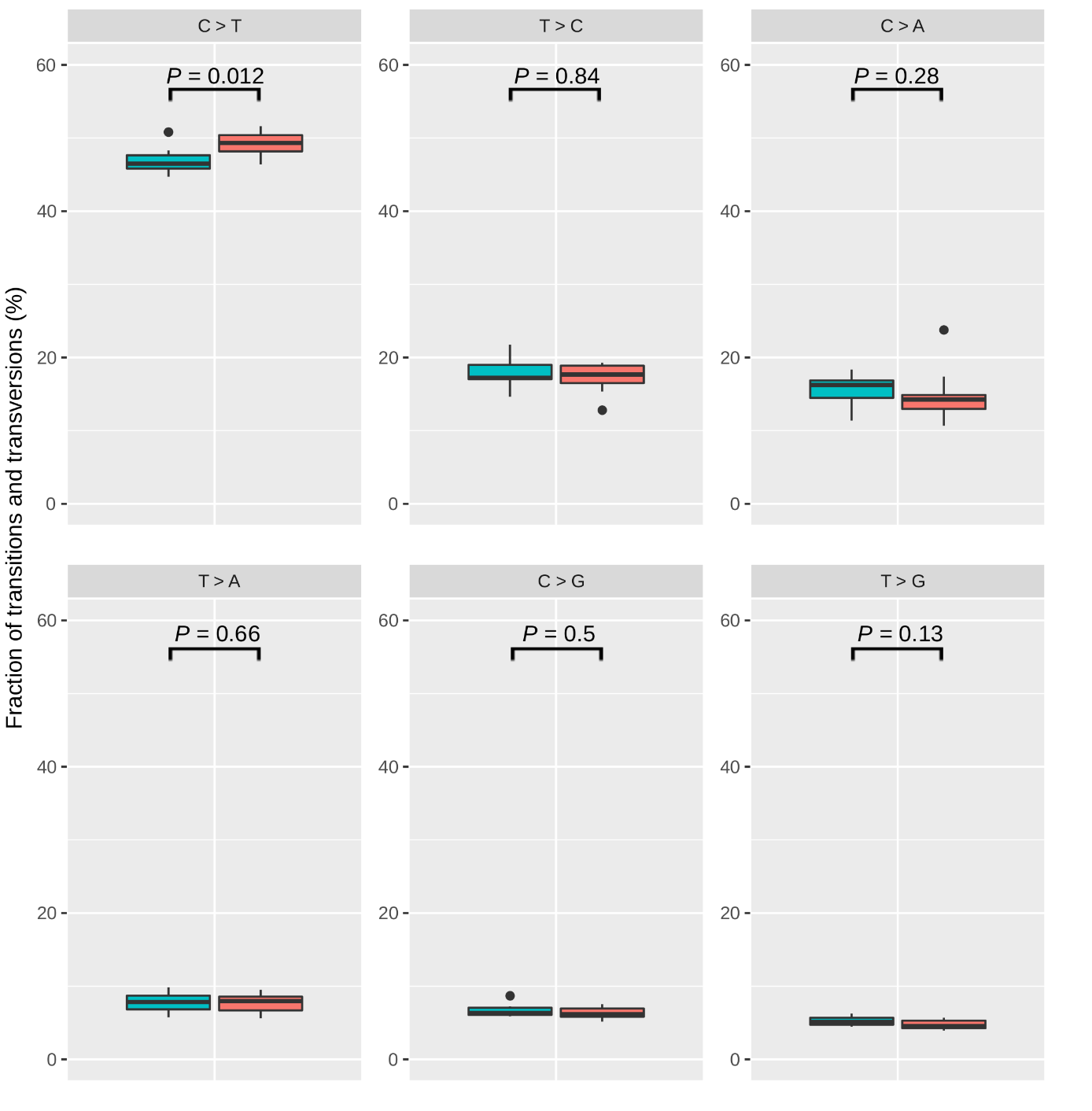
**

Comparison of the frequencies of the different transition and transversion types in *RUNX1*::*RUNX1T1*-positive (blue boxes) and *CBFB*::*MYH11*-positive (red boxes) acute myeloid leukemia. Except for C>T, which was slightly more common in the cases with *CBFB*::*MYH11*, the frequencies of the different single nucleotide variants did not differ between the two gene fusion groups. The *P*-values are based on Mann Whitney U-test analyses.

**Supplementary Figure 2**

**
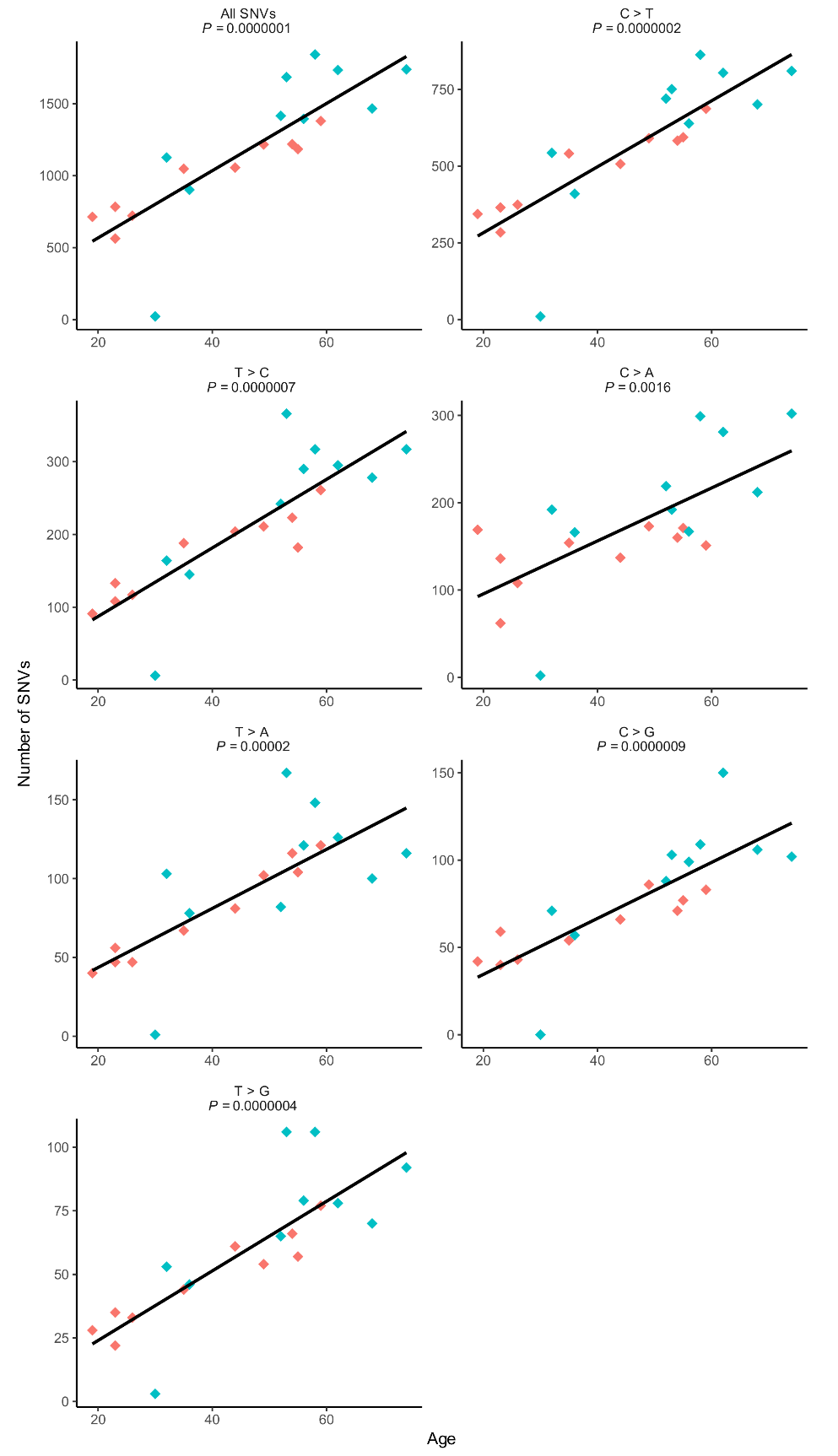
**

Total number of single nucleotide variants (SNVs) in relation to age and correlations between the different transition/transversion types and age. The *RUNX1*::*RUNX1T1*- and *CBFB*::*MYH11*-positive cases are shown in blue and red, respectively. The *P*-values are based on linear regression analyses.

**Supplementary Figure 3**

**
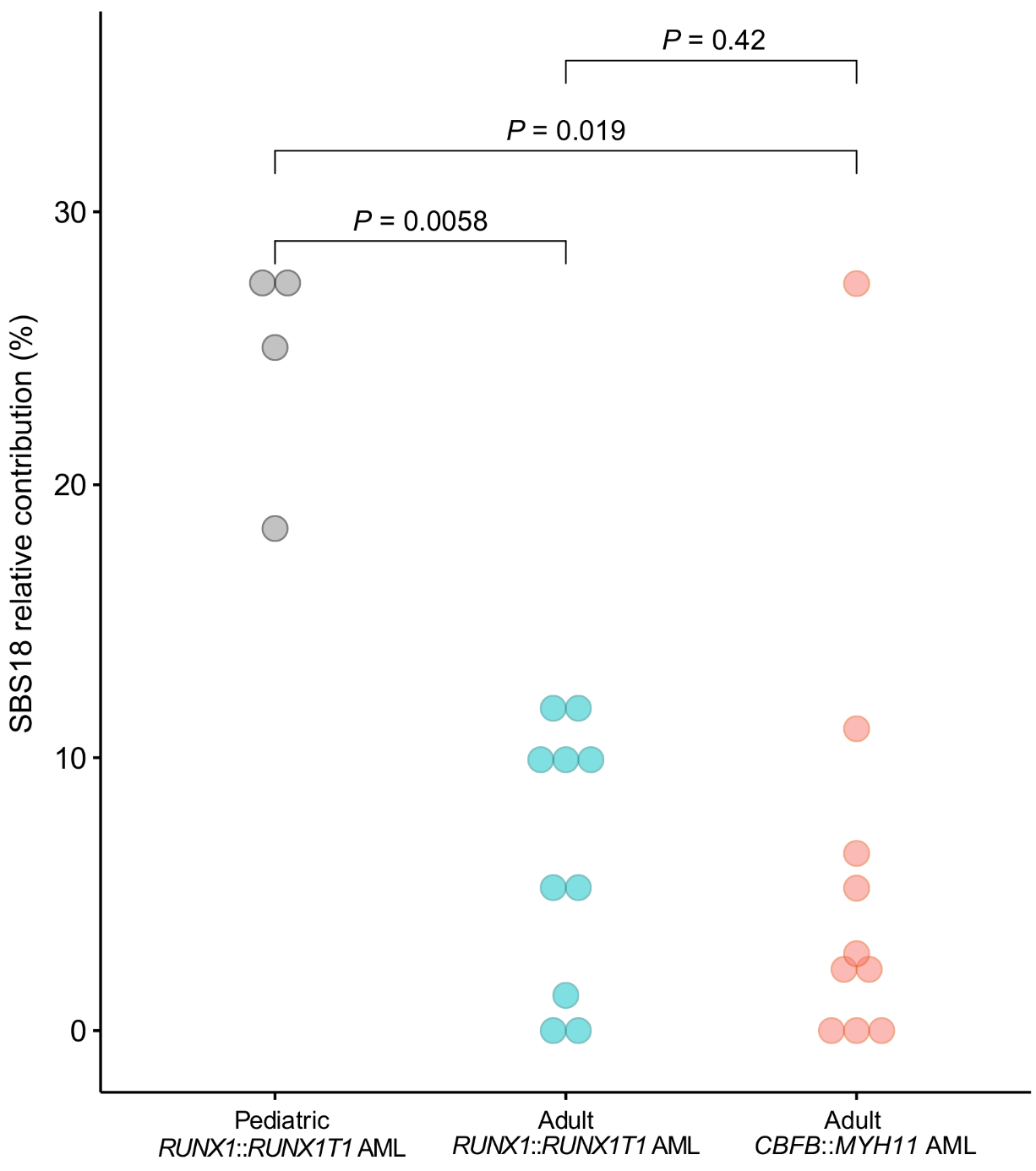
**

Comparison of the relative contribution of the single base substitution 18 (SBS18) signature in adult *RUNX1*::*RUNX1T1*- and *CBFB*::*MYH11*-positive cases and in pediatric cases with *RUNX1*::*RUNX1T1* fusion.

**Supplementary Figure 4**

**
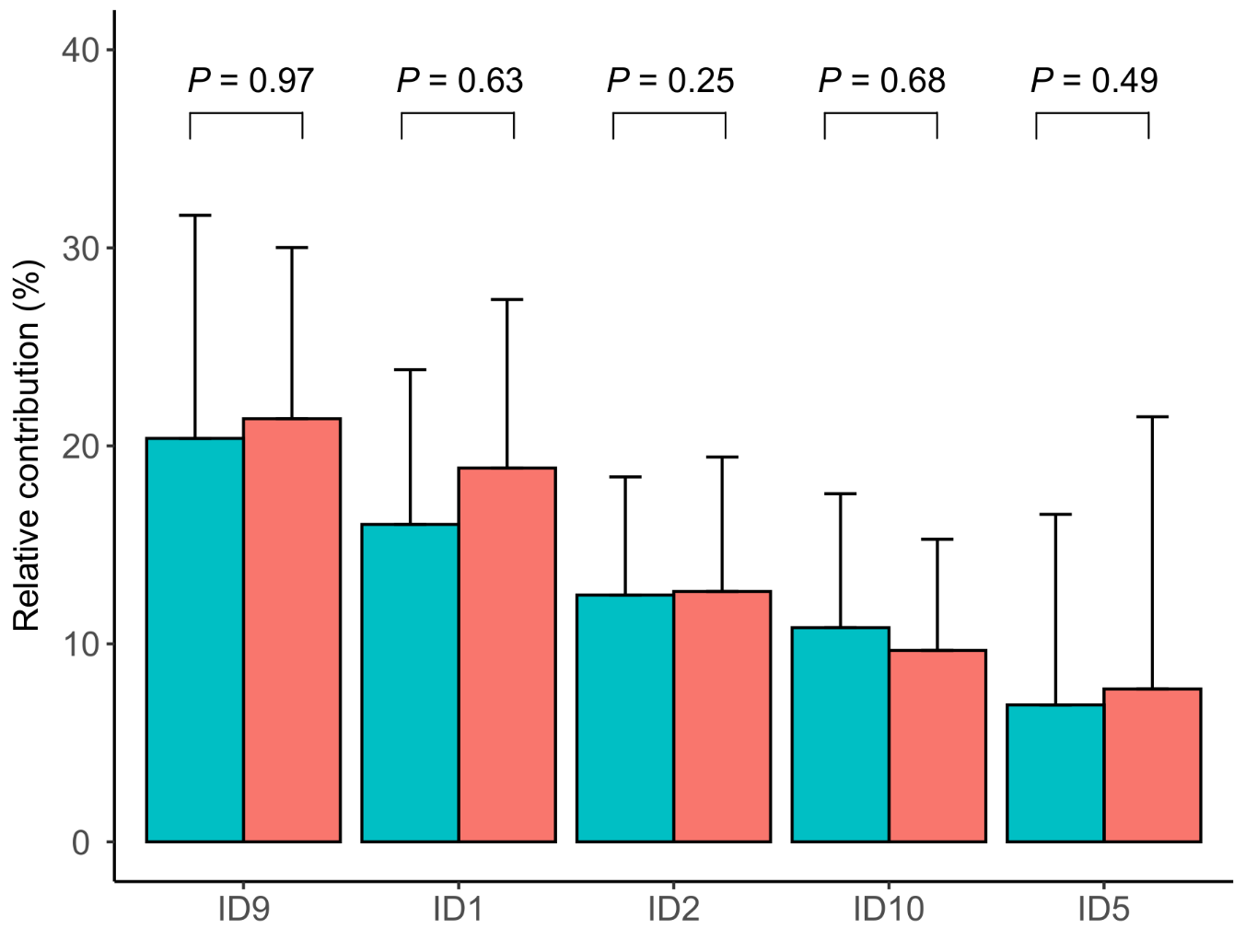
**

Relative contributions of the five most common insertion/deletion (ID) signatures in adult core binding factor acute myeloid leukemia, as determined by the sum of the relative contribution of each signature in each case within each subgroup. The *RUNX1*::*RUNX1T1*- and *CBFB*::*MYH11*-positive cases are shown in blue and red, respectively. The *P*-values are based the Mann-Whitney U test analyses.
